# Muscle Mitochondrial DNA Copy Number, Deletion Mutation Frequency, and Physical Performance in Older Adults

**DOI:** 10.1093/geroni/igaa057.3279

**Published:** 2020-12-16

**Authors:** Jonathan Wanagat, Allen Herbst, Steven Prior, Judd Aiken, Debbie McKenzie, Nianjun Liu, Xiwei Chen, David Allison

**Affiliations:** 1 UCLA, Los Angeles, California, United States; 2 University of Alberta, Edmonton, Alberta, Canada; 3 University of Maryland School of Medicine, Baltimore, United States; 4 University of Alberta-Edmonton, Edmonton, Canada; 5 Indiana University, Bloomington, Indiana, United States

## Abstract

Mitochondrial DNA (mtDNA) quantity and quality influence hallmarks of aging – mitochondrial dysfunction and genomic instability. The interactions between mtDNA quantity and quality and physical performance have not been extensively examined in humans. The aim of this study was to test the interactions between skeletal muscle mtDNA copy number, mtDNA deletion mutation frequency, and physical performance measures in older adults. Total DNA was isolated from muscle biopsies and used for quantitation of mtDNA copy number and mutation frequency by digital PCR. The biopsies were obtained from a cross-sectional cohort of 53 adults aged 50 to 86 years. Before the biopsy, physical performance measures were collected. MtDNA deletions increased exponentially with advancing age. On average, mtDNA deletion frequency increased 18-fold between 50 and 80, with a trend toward lower deletion frequency in females. MtDNA deletion frequency predicted declines in VO2 max, where 4.7% of the variation in VO2 max was explained by mtDNA deletion frequency. MtDNA copy number was negatively correlated with age and mtDNA deletion frequency, but positively correlated with lean mass. There was a trend to lower mtDNA deletion frequency in females, consistent with increased longevity in females. Larger studies may better delineate sex effects. These data are consistent with a role for mitochondrial function and genome integrity in the maintenance of physical performance with age. Analyses of mtDNA quality and quantity in longitudinal studies could extend our understanding of the importance of mitochondria in human aging and longevity.

